# Association of metabolic syndrome and its components with Parkinson’s disease: a cross-sectional study

**DOI:** 10.1186/s12902-024-01623-3

**Published:** 2024-06-19

**Authors:** Yue Shi, XueYi Zhang, Yue Feng, ZongXiang Yue

**Affiliations:** 1 Chengdu University of Traditional Chinese Medicine, Chengdu, China; 2 Meishan Hospital of Traditional Chinese Medicine, Meishan, China

**Keywords:** Metabolic syndrome, Parkinson’s Disease, Hypertension, Diabetes Mellitus

## Abstract

**Background:**

The interrelation between metabolic syndrome (MetS) and Parkinson’s disease (PD) likely arises from shared pathological mechanisms. This study thus aims to examine the impact of MetS and its components on PD.

**Methods:**

This study utilized data extracted from the National Health and Nutrition Examination Survey database spanning 1999 to 2020. The random forest algorithm was applied to fill in the missing data. Propensity score optimal full matching was conducted. The data were adjusted by total weights derived from both sampling and matching weights. The weighted data were utilized to create multifactor logistic regression models. Odds ratios (ORs) and average marginal effects, along with their corresponding 95% confidence intervals (CIs), were calculated.

**Results:**

MetS did not significantly affect the risk of PD (OR: 1.01; 95% CI: 0.77, 1.34; *P* = 0.92). Hypertension elevated the risk of PD (OR: 1.33; 95% CI: 1.01, 1.76; *P* = 0.045), accompanied by a 0.26% increased probability of PD occurrence (95% CI: 0.01%, 0.52%; *P* = 0.04). Diabetes mellitus (DM) had a 1.38 times greater likelihood of developing PD (OR:1.38; 95% CI: 1.004, 1.89; *P* = 0.046), corresponding to a 0.32% increased probability of PD occurrence (95% CI: -0.03%, 0.67%; *P* = 0.07). Nevertheless, no correlation was observed between hyperlipidemia, waist circumference and PD.

**Conclusion:**

MetS does not affect PD; however, hypertension and DM significantly increase the risk of PD.

**Supplementary Information:**

The online version contains supplementary material available at 10.1186/s12902-024-01623-3.

## Introduction

Metabolic syndrome (MetS) encompasses the accumulation of excessive abdominal fat, insulin resistance (IR), abnormal lipid levels, and high blood pressure [1, 2]. Quantifying the prevalence of MetS is challenging due to the lack of global statistics. Nevertheless, given that MetS occurs at a rate approximately three times higher than diabetes, its prevalence might be estimated at approximately 25% of the global population [3]. Parkinson’s disease (PD) is projected to experience a fourfold increase in frequency over the next 30 years, becoming the second most prevalent neurodegenerative illness [4]. PD is characterized by clinical manifestations [5] such as bradykinesia, stiffness, a flexed posture, “freezing” episodes, and the loss of postural reflexes. These motor abnormalities, commonly observed in PD, result from the depletion of dopamine neurons in the brain’s nigrostriatal pathway.

Despite the elusive nature of PD pathophysiology, emerging evidence suggests a complex involvement of MetS in the disease’s pathological processes. These processes encompass IR, neuroinflammation, accumulation of α-synuclein proteins, and mitochondrial dysfunction [6]. A chronic hyperglycemic state may lead to changes in postsynaptic dopamine receptors, as suggested by research on rat models [7]. Obesity-related low-grade inflammation can induce neuroinflammation through various pathways, including the choroid plexuses and disruption of the blood–brain barrier. Therefore, we hypothesized that metabolic and neurodegenerative diseases may be interrelated because of their shared pathophysiological mechanisms. Although several studies have investigated the association between MetS components and PD events, the results have been inconsistent [8] due to varying methodologies and diverse study populations. Establishing whether MetS and its components are independent risk factors for PD is crucial to determining if patients newly diagnosed with MetS should be monitored for PD development and if PD patients might already exhibit concurrent metabolic abnormalities. This study further explores the impact of MetS and its components on PD utilizing the National Health and Nutrition Examination Survey (NHANES) database, which offers a nationally representative sample.

## Materials and methods

### Study population

Data was obtained from the NHANES, a comprehensive study that investigates a wide range of demographic characteristics and health issues in the United States. The survey sample is selected from all states across the country, ensuring national representation. Due to the age of onset of PD, the study included individuals who were 18 years old or older. And since the special characteristics of pregnant women, they were not included in this study. Cases with missing data for MetS and PD were excluded from the analysis. Data from eleven consecutive two-year cycles, spanning from 1999 to 2000 to 2019–2020 (1999–2000, 2001–2002, 2003–2004, 2005–2006, 2007–2008, 2009–2010, 2011–2012, 2013–2014, 2015–2016, 2017–2018, and 2019–2020), were utilized. The NHANES study received approval from the National Center for Health Statistics Ethics Review Board, with the most recent review conducted on August 24, 2022. Informed consent was obtained from all participants.

### MetS diagnosis

In this study, the revised diagnostic criteria [9] were determined through consultation between the American Heart Association/National Heart, Lung, and Blood Institute and the International Diabetes Federation. The diagnostic criteria consist of the following five elements: (i) a waist circumference of at least 102 cm for men and 88 cm for women in the U.S. population; (ii) elevated triglycerides of at least 150 mg/dL; (iii) decreased high-density lipoprotein (HDL) cholesterol of at least 40 mg/dL for men and 50 mg/dL for women; (iv) systolic blood pressure of at least 130 mmHg and/or diastolic blood pressure of at least 85 mmHg; (v) fasting blood glucose of 100 mg/dL or higher. Drug treatment serves as an alternative indication for the last four elements. If any three of these criteria are met, a diagnosis of MetS may be made.

MetS was categorized into four components: high waist circumference (WC), hypertension, diabetes mellitus (DM), and hyperlipidemia. The diagnosis is based on the presence of each condition and is not limited to a specific indicator under the MetS diagnosis entry. The diagnosis of high WC was the same as the first item of the MetS diagnoses. Hypertension was diagnosed based on the following factors: the mean of three blood pressure readings [≥ 140 mmHg for systolic blood pressure and ≥ 90 mmHg for diastolic blood pressure], antihypertensive drug use, and self-reported high blood pressure. DM diagnosis was based on self-report of diabetes, glucose-lowering medication usage, levels of glycated hemoglobin [≥ 6.5 mmol/L], fasting glucose [≥ 7 mmol/L], random glucose [≥ 11.1 mmol/L], and oral glucose tolerance test [≥ 11.1 mmol/L]. Hyperlipidemia was characterized by hypertriglyceridemia [triglycerides ≥ 150 mg/dL], hypercholesterolemia [total cholesterol ≥ 200 mg/dL, low-density lipoprotein ≥ 130 mg/dL, or HDL < 40 mg/dL for men and 50 mg/dL for women], and the use of lipid-lowering medications.

### PD diagnosis

PD was diagnosed based on drug use recorded in the drug file provided by the NHANES database. This study focuses on the second level category called antiparkinson agents according to the Multum Lexicon Therapeutic Classification Scheme. Within this category, two sub-level three level categories are considered: anticholinergic antiparkinson agents and dopaminergic antiparkinsonism agents.

### Covariates

Age, sex, race, education, smoking, alcohol and coffee consumption [10] were self-reported by participants. Smoking status was categorized into three groups: non-smokers [< 100 cigarettes in a lifetime], former smokers [≥ 100 cigarettes in a lifetime but not currently smoking], and current smokers [≥ 100 cigarettes in a lifetime and smoking some days or every day]. Alcohol consumption [11] was divided into three categories: non-alcohol user [did not drink last year or had 1 drink for male], mild alcohol user [≥ 1 drinks for females and 2 for males, or binge drinking (≥ 4 drinks on the same occasion for females, ≥ 5 drinks on the same occasion for males) ≥ 2 and < 5 days per month, and heavy alcohol user [≥ 3 drinks for females and 4 drinks for males, or binge drinking on 5 or more days per month]. Coffee consumption refers to the amount of coffee (in grams) consumed from dietary data on the first day. For simplicity in subsequent analysis, the data were categorized into three levels: Q1, Q2, and Q3, with corresponding medians of 0, 179.9, and 503.2 g.

### Statistical analysis

The missing data for the two primary variables, MetS and PD, were excluded from the analysis. Missing values for the remaining variables were imputed using the random forest algorithm, which employs known variables as independent variables and variables with missing values as dependent variables to predict the missing values. This method offers high prediction accuracy and robustness. To balance the effects of covariates between groups with and without MetS, including its components, the propensity score matching (PSM) approach was utilized. Given the limited number of PD patients, optimal full matching was chosen to minimize sample loss. Sampling weights were calculated before matching. These weights were generated for complex sample designs using mobile examination center exam weights (WTMEC4YR for 1999–2002 and WTMEC2YR for 2003–2020), along with the variables SDMVPSU and SDMVSTRA. The matching process took into account the sampling weight [12–14]. The total weight was calculated by multiplying the sampling and matching weight. The data was then weighted using the total weight. The standardized mean difference (SMD) was the statistical measure used to assess balance, with a value of < 0.1 indicating equilibrium. Continuous variables were described using mean and standard error, while categorized variables were described using frequency and percentage. The balance of covariates was re-evaluated by independent-samples t-test and chi-square test. The study estimated the effect of MetS and its component on PD in the weighted sample by total weights, adjusting for the covariates to improve precision and reduce bias. Simultaneously, a stratification analysis of variables that failed to match balance was conducted. Effect sizes were expressed as odds ratios (ORs) and the average marginal effects with a 95% confidence interval (CI). The significance level for two-tailed tests was set at 0.05. A sensitivity analysis was performed using the conventional method of intervariable adjustment, multi-factor logistic regression, with data weighted by sampling weights. It is important to note that the results of the analysis of the four components of MetS should be interpreted as exploratory due to the potential for type 1 errors from multiple comparisons. This research was analyzed using R software (version 4.2.2, R Development Core Team. Vienna, Austria) with the following packages: missForest (version 1.5) for imputation, MatchIt (version 4.5.0) for PSM, cobalt (version 4.5.1) for assessing post-PSM data balance, survey (version 4.1-1) for weighting and logistic regression, marginaleffects (version 0.15.1) for estimating average marginal effects, and ggplot2 (version 3.4.2) and cowplot (version 1.1.1) for visualization.

## Results

### Population characteristics

**Fig. 1:**
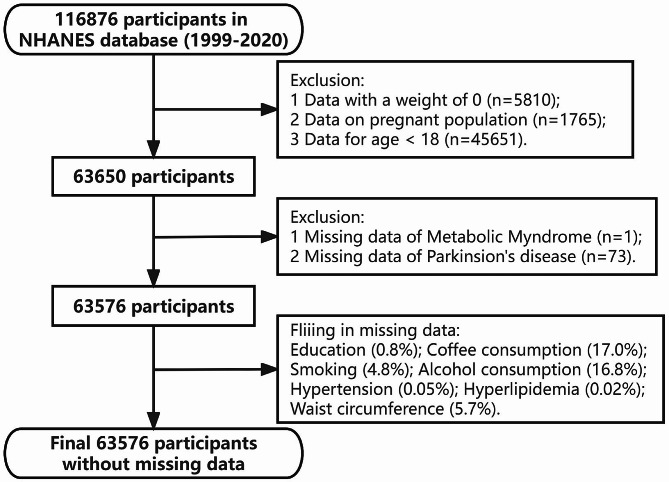
Flow-chart for the participants’ selection

The study included data of 63,576 individuals after applying filters, including 591 participants with PD. The data was weighted to represent the estimated population of the United States, which is approximately 223,670,260. The specific screening procedure is illustrated in Fig. 1. The population with MetS had a higher prevalence of males, advanced age, and greater levels of coffee intake. The MetS group consisted of a significant proportion of former smokers and light drinkers while having a lower proportion of current smokers and heavy drinkers than those without MetS. Table 1 presents the characteristics of the populations with and without MetS. After performing PSM, differences were noted in sex, alcohol and coffee consumption between those with and without MetS, as shown in Supplementary Material Table S1. The baseline characteristics of hypertension, hyperlipidemia, DM, and different WC after PSM are shown in Tables S2-S5 of the Supplementary Material.

**Table 1:** Comparison of baseline characteristics in participants with and without metabolic syndrome before propensity score matching

variable	Total (*n* = 63,576)	Non-MetS (*n* = 45,029)	MetS (*n* = 18,547)	*P* value
Age	46.45(0.17)	42.62(0.17)	56.36(0.18)	< 0.0001
Sex				< 0.0001
Female	32,060(51.15)	22,791(51.77)	9269(49.55)	
Male	31,516(48.85)	22,238(48.23)	9278(50.45)	
BMI	28.74(0.06)	27.54(0.06)	31.84(0.08)	< 0.0001
Coffee (gram)	310.86(3.86)	292.72(4.17)	357.78(5.31)	< 0.0001
Race				< 0.0001
Mexican American	10,952( 8.29)	7915(8.73)	3037(7.14)	
Non-Hispanic Black	14,227(11.30)	9989(11.34)	4238(11.21)	
Non-Hispanic White	26,460(67.29)	18,129(65.77)	8331(71.21)	
Others	11,937(13.12)	8996(14.16)	2941(10.43)	
Education				< 0.0001
High school or equivalent	25,597(36.72)	18,308(36.33)	7289(37.72)	
College or above	30,864(57.52)	22,170(58.38)	8694(55.30)	
Less than high school	7115( 5.76)	4551(5.29)	2564(6.98)	
Smoking				< 0.0001
Never	36,166(55.01)	26,603(56.70)	9563(50.65)	
Former	14,640(24.02)	8686(20.61)	5954(32.83)	
Now	12,770(20.97)	9740(22.69)	3030(16.51)	
Alcohol consumption				< 0.0001
No	20,264(25.15)	13,992(24.16)	6272(27.70)	
Mild	30,135(52.48)	20,910(51.51)	9225(54.99)	
Heavy	13,177(22.37)	10,127(24.33)	3050(17.31)	
Parkinson’s disease				< 0.0001
No	62,985(99.11)	44,675(99.25)	18,310(98.77)	
Yes	591(0.89)	354(0.75)	237(1.23)	

MetS: metabolic syndrome. The results presented in the table are all weighted by the sample weights (except for the frequency)

### Balance test for PSM

**Fig. 2:**
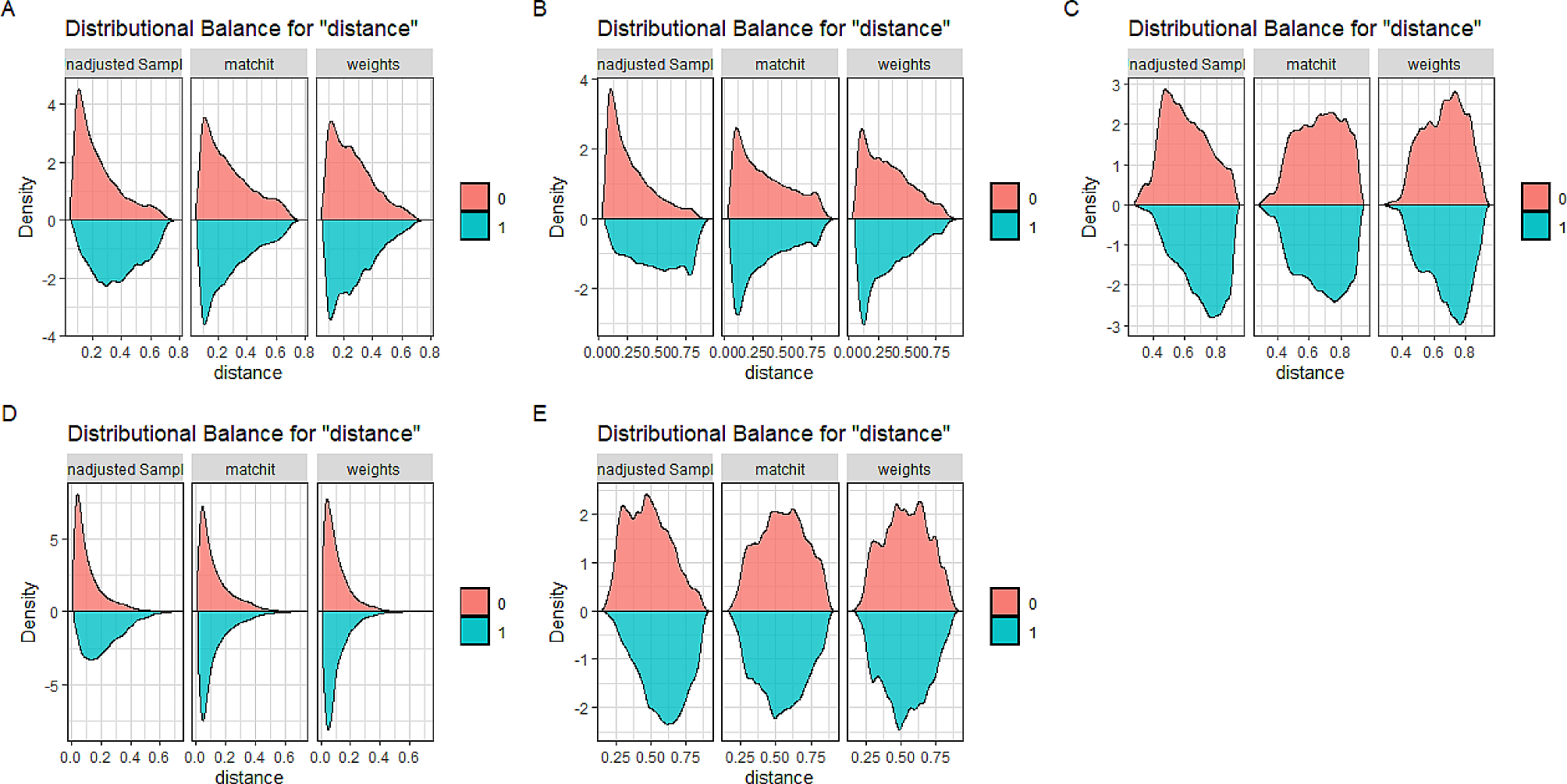
Density plots before and after propensity score matching. **A**: Metabolic syndrome, **B**: Hypertension, **C**: Hyperlipidemia, **D**: Diabetes mellitus, **E**: Waist circumference

For the data weighted by total weight, the balance statistic SMD was calculated, and the balance of covariates was achieved between individuals with and without MetS or its components (SMD < 0.1), as shown in Supplementary Material Table S6. Figure 2 demonstrates the balance of the data for PS before matching, after matching with matching weights weighted and after matching with total weights.

### Association between MetS and PD

The presence of MetS did not significantly impact the risk of PD (OR: 1.01; 95% CI: 0.77, 1.34; *P* = 0.92), as seen in Table 2. Conversely, DM increased the likelihood of developing PD (OR:1.38; 95% CI: 1.004, 1.89; *P* = 0.047). DM was associated with a slight increase of 0.32% (95% CI: -0.03%, 0.67%; *P* = 0.07) in the likelihood of developing PD. Individuals with hypertension had a 1.33-fold increased likelihood of developing PD compared with those without hypertension (OR: 1.33; 95% CI: 1.006, 1.76; *P* = 0.045). Hypertension was associated with a 0.26% (95% CI: 0.01%, 0.52%; *P* = 0.04) increase in the likelihood of developing PD. Nevertheless, there was no correlation between hyperlipidemia and WC and PD. Logistic regression analyses in sensitivity analyses suggested that hypertension (OR: 1.38; 95% CI: 1.03, 1.847; *P* = 0.03), DM (OR: 1.36; 95% CI: 1.07, 1.74; *P* = 0.01), and high WC (OR: 1.44; 95% CI: 1.13, 1.84; *P* = 0.003) increased the odds of developing PD, as shown in Table 3.

**Table 2:** Relationship of metabolic syndrome and its components with Parkinson’s disease after propensity score matching

Exposure	OR (95% CI)	*P* value	AME (95% CI)	*P* value
Metabolic syndrome	1.01 (0.77,1.34)	0.923	0.01% (-0.25%,0.27%)	0.923
Diabetes mellitus	1.38 (1.004,1.89)	0.047	0.32% (-0.03%,0.67%)	0.073
Hypertension	1.33 (1.006,1.76)	0.045	0.26% (0.008%,0.52%	0.043
Hyperlipidemia	0.88 (0.65,1.21)	0.442	-0.12% (-0.42%,0.19%)	0.457
Waist circumference	1.29 (0.97,1.73)	0.085	0.24% (-0.02%,0.49%)	0.071

AME: average marginal effect

**Table 3:** Relationship of metabolic syndrome and its components with Parkinson’s disease without propensity score matching

Exposure	Crude			Model†	
	OR (95% CI)	*P*		OR (95% CI)	*P*
Metabolic syndrome	1.65(1.30,2.10)	< 0.0001		1.05(0.81,1.35)	0.713
Prediabetes	1.98(1.30,3.02)	0.002		1.48(0.97,2.26)	0.072
Diabetes mellitus	2.27(1.79,2.87)	< 0.0001		1.44(1.12,1.85)	0.005
Hyperlipidemia	1.64(1.25,2.16)	< 0.001		1.06(0.80,1.41)	0.679
Hypertension	2.52(1.98,3.20)	< 0.0001		1.38(1.03,1.85)	0.030
Waist circumference	1.99(1.57,2.51)	< 0.0001		1.44(1.13,1.84)	0.003

†: Multifactor logistic regression model adjusting for age, sex, race, education, smoking, alcohol and coffee consumption

The results of the stratified analyses are shown in Supplementary Material Table S7. Among individuals with high coffee consumption, hypertension (OR: 1.94; 95% CI: 1.25, 3.02; *P* = 0.003) and DM (OR: 1.79; 95% CI: 1.07, 2.98; *P* = 0.025) were associated with an increased risk of PD. High WC was linked to an increased risk of PD in individuals younger than 50 years (OR: 2.15; 95% CI: 1.28, 3.60; *P* = 0.004) and those with lower education (OR: 1.99; 95% CI: 1.003, 3.74; *P* = 0.049).

## Discussion

MetS is a widespread and escalating global public health issue associated with multiple chronic illnesses. The study aimed to investigate the relationship between MetS and PD, which are believed to share overlapping pathological processes. However, this study did not find any correlation between MetS and the likelihood of developing PD. When examining the components of MetS, it was revealed that high WC and hyperlipidemia had no impact on PD, while hypertension and DM increased the likelihood of PD.

Previous research has presented contradictory findings regarding the relationship between MetS and PD. Some studies suggest that MetS increases the risk of PD, while others propose the opposite, and some find no significant relationship [15]. MetS, being a composite comprised of various states, produces distinct effects from each component. For example, a study [16] using data from the National Health Insurance Service of Korea found that the incidence of PD was 1.23 times higher in individuals with MetS. High blood pressure, low HDL cholesterol, and high fasting blood glucose were linked to an increased incidence of PD, but not high WC. These findings [17, 18] remained consistent even when considering the length of the follow-up period or the longitudinal research. Additionally, abdominal obesity was associated with an elevated risk of developing PD. MetS was found to increase the risk of progression from mild Parkinsonian signs to PD [19]. MetS exacerbates non-motor symptoms in patients with PD [20], such as cognitive impairment [21]. One study [22] suggests that hypertension exerts a detrimental effect on memory and verbal fluency in early PD. According to a vast Finnish investigation [23], elevated total cholesterol levels were correlated with an increased risk of PD. A meta-analysis [24] revealed a 27% higher relative risk of PD in individuals with DM than in those without. Antihypertensive medication may reduce the risk of PD in patients with newly diagnosed hypertension [25, 26]. Nevertheless, the MetS population has a reduced risk of PD. While elevated serum triglyceride and fasting glucose levels were predictive of a lower incidence of PD, a higher BMI was suggestively associated with an increased risk of PD [27]. MetS was associated with reduced occurrence of falls in patients with PD, indicating a potential beneficial effect on motor symptoms [28]. The results of a Japanese case-control study showed a significant link between DM, hypertension, and high cholesterol levels and a lower risk of PD [29]. Additional studies have demonstrated that a high WC [30] and obesity [31] were linked to a reduced likelihood of PD. Furthermore, a study [32] also found no association between a prior medical history of hypertension, hypercholesterolemia, or diabetes with the chance of developing PD.

Numerous pathogenic processes, including IR, oxidative stress, immunology, and inflammation, may overlap between MetS and PD [8, 33, 34]. This overlap suggests that PD should be treated as a metabolic disease [35, 36]. IR is a key factor in type 2 diabetes and is now recognized as an important mechanism in the association between DM and PD. Insulin, beyond its role in glucose regulation, acts as a major contributor to many diseases [37], including neurological conditions, and plays various functions in the brain [38], including the maintenance of cellular homeostasis, prevention of ROS generation, and promotion of cell survival. The brain is highly responsive to insulin, enabling it to regulate the functions of neurons and glial cells, resulting in changes in emotions, cognition, and behavior [39]. When insulin function in the brain is compromised, it initiates cellular disturbances, resulting in pathological states. For example, MetS and DM can render dopaminergic neurons vulnerable [40] and affect dopamine neuron survival [41]. IR may lead to reduced expression of dopamine transporter proteins on the striatal surface, as demonstrated in animal models [42]. Furthermore, the development of PD may be influenced by mitochondrial dysfunction, oxidative stress, and microglia-mediated inflammatory responses, all of which could contribute to the association between PD and DM.

Hypertension may increase the risk of PD through several mechanisms [43, 44]. First, hypertension induces dysregulation of the autonomic nervous system, leading to an increased cardiac load and rapid blood flow in the arteries, exerting variable degrees of pressure on the cerebrovascular system. Patients with hypertension often experience blood pressure fluctuations, especially at night, resulting in an imbalance in cerebral perfusion, particularly at the level of small arteries and microvessels. The imbalance created by hypertension can lead to an increased risk of neuronal damage, further heightening the likelihood of developing PD. Additionally, arterial stiffness induced by hypertension reduces the elasticity of cerebral blood vessels, leading to increased resistance to cardiac output and subsequently diminishing cerebral perfusion. Furthermore, hypertension can disrupt the blood-brain barrier and cause capillary abnormalities, both of which contribute to accelerated neuronal degeneration, ultimately increasing the risk of PD.

Cholesterol has both preventive and detrimental effects on PD neuropathology [45]. Cholesterol may have potential neuroprotective benefits against the development of PD by modulating ion channels and receptors, which can be influenced by changes in cholesterol levels. However, elevated blood cholesterol levels can indirectly increase the risk of PD. This is due to the induction of oxidative stress, inflammation, and apoptosis caused by a compound called 27-hydroxycholesterol. Furthermore, the presence of cholesterol is associated with the degeneration of dopaminergic neurons in the substantia nigra and the aggregation of α-synuclein in the brain, both of which are factors contributing to the development of PD.

This study incorporates data spanning 11 year-cycles, from 1999 to 2020, extracted from the NHANES database, which represents a substantial study population. To ensure an accurate representation of nearly the entire population of the United States, the data is weighted using a complex sampling procedure, even when performing PSM. Additionally, our study provides two distinct measures of effect. Given the low positivity rate of PD, the numerical magnitude of the OR can be somewhat misleading. Hence, we also offer estimates of the average marginal effect, which is equivalent to the risk difference in this study. This dual approach enhances the comprehensiveness of our findings, allowing readers to better understand the true effect size between variables. However, there are certain unavoidable limitations in this study. It is a cross-sectional study, and while we used PSM and traditional covariate adjustment methods to mitigate the influence of confounding factors, the presence of unknown variables and the potential for reverse causation remain. Therefore, the results of this study do not support the inference of causality. Furthermore, due to the absence of data regarding the symptoms of PD in the database, this study solely relied on the presence or absence of relevant medication to diagnose PD. Consequently, it was unable to gather information on the symptoms of PD in the subset of patients who were not taking medication. The results of the present study suggest that MetS is not associated with PD, whereas DM and hypertension are correlated with PD. Due to the limitations of the research methodology of this study, further validation through additional studies is warranted. Future research should include prospective cohort studies and Mendelian randomization studies to investigate causality, as well as mechanistic studies to elucidate potential pathways.

## Conclusion

The findings do not support the hypothesis that MetS plays a direct role in influencing the development of PD. However, within the multifaceted landscape of MetS, it is evident that hypertension and DM are associated with an increased likelihood of developing PD. On the other hand, high WC and hyperlipidemia do not seem to have a significant impact on the risk of PD.

### Electronic supplementary material

Below is the link to the electronic supplementary material.

Supplementary Material 1

## Data Availability

The original data for this study are available from publicly available databases (https://www.cdc.gov/nchs/nhanes/index.htm). Further inquiries can be directed to the corresponding author.

## Abbreviations

MetS: Metabolic Syndrome
IR: Insulin resistance
PD: Parkinson’s disease
NHANES: National Health and Nutrition Examination Survey
HDL: High-density lipoprotein
WC: Waist circumference
DM: Diabetes mellitus
PSM: Propensity score matching
SMD: Standardized mean difference
OR: Odds ratio
CI: Confidence interval
