# Supplementary material for: Association of metabolic syndrome and its components with Parkinson’s disease: a cross-sectional study
Source: BMC Endocr Disord. 2024 Jun 19;24:92. doi: 10.1186/s12902-024-01623-3 (PMC11186221; doi:10.1186/s12902-024-01623-3)
Supplement: Supplementary file 1 — Supplementary Material 1 [file 12902_2024_1623_MOESM1_ESM.docx]

**Supplementary Material**

Supplementary Table 1. Comparison of baseline characteristics in participants with and without diabetes mellitus after propensity score matching.

|  | Total | Non-DM | DM | *P* value |
| --- | --- | --- | --- | --- |
| Age | 46.69(0.12) | 46.68(0.10) | 46.77(0.51) | 0.85 |
| Sex |  |  |  | 0.01 |
| Female | 32060(51.74) | 26762(51.09) | 5298(55.02) |  |
| Male | 31516(48.26) | 25964(48.91) | 5552(44.98) |  |
| BMI | 29.16(0.07) | 28.17(0.04) | 34.14(0.34) | < 0.0001 |
| Coffee (gram) | 314.53(3.19) | 315.20( 2.59) | 311.14(14.16) | 0.78 |
| Race |  |  |  | 0.01 |
| Mexican American | 10952( 7.96) | 8909(8.19) | 2043(6.80) |  |
| Non-Hispanic Black | 14227(11.17) | 11404(11.17) | 2823(11.15) |  |
| Non-Hispanic White | 26460(67.95) | 22662(67.52) | 3798(70.15) |  |
| Others | 11937(12.92) | 9751(13.13) | 2186(11.90) |  |
| Education |  |  |  | 0.44 |
| High school or equivalent | 25597(36.43) | 21230(36.66) | 4367(35.29) |  |
| College or above | 30864(57.83) | 26416(57.61) | 4448(58.95) |  |
| Less than high school | 7115( 5.73) | 5080(5.73) | 2035(5.76) |  |
| Smoking |  |  |  | 0.27 |
| Never | 36166(54.66) | 30644(54.82) | 5522(53.86) |  |
| Former | 14640(24.09) | 11055(24.26) | 3585(23.21) |  |
| Now | 12770(21.26) | 11027(20.92) | 1743(22.93) |  |
| Alcohol consumption |  |  |  | 0.32 |
| No | 20264(25.22) | 15650(25.14) | 4614(25.64) |  |
| Mild | 30135(52.27) | 25241(52.63) | 4894(50.45) |  |
| Heavy | 13177(22.51) | 11835(22.23) | 1342(23.91) |  |

DM: Diabetes mellitus. The results presented in the table are all weighted by total weights of combining sampling and matching weights (except for the frequency).

Supplementary Table 2. Comparison of baseline characteristics in participants with and without hypertension after propensity score matching.

|  | Total | Non-Hypertension | Hypertension | *P* value |
| --- | --- | --- | --- | --- |
| Age | 46.41(0.14) | 46.59(0.14) | 46.15(0.27) | 0.16 |
| Sex |  |  |  | 0.02 |
| Female | 32060(49.37) | 19190(50.15) | 12870(48.18) |  |
| Male | 31516(50.63) | 19016(49.85) | 12500(51.82) |  |
| BMI | 29.12(0.05) | 27.65(0.05) | 31.34(0.11) | < 0.0001 |
| Coffee (gram) | 308.46(2.96) | 313.71(3.30) | 300.52(5.47) | 0.04 |
| Race |  |  |  | 0.81 |
| Mexican American | 10952( 8.23) | 7563(8.27) | 3389(8.17) |  |
| Non-Hispanic Black | 14227(11.01) | 7390(10.86) | 6837(11.24) |  |
| Non-Hispanic White | 26460(67.82) | 15362(67.88) | 11098(67.73) |  |
| Others | 11937(12.94) | 7891(12.99) | 4046(12.86) |  |
| Education |  |  |  | 0.12 |
| High school or equivalent | 25597(36.11) | 15275(36.60) | 10322(35.37) |  |
| College or above | 30864(58.16) | 19366(57.58) | 11498(59.02) |  |
| Less than high school | 7115( 5.73) | 3565(5.82) | 3550(5.61) |  |
| Smoking |  |  |  | 0.96 |
| Never | 36166(54.83) | 23320(54.74) | 12846(54.96) |  |
| Former | 14640(23.83) | 6831(23.87) | 7809(23.78) |  |
| Now | 12770(21.34) | 8055(21.39) | 4715(21.26) |  |
| Alcohol consumption |  |  |  | 0.66 |
| No | 20264(24.88) | 10846(24.92) | 9418(24.81) |  |
| Mild | 30135(52.58) | 17950(52.78) | 12185(52.28) |  |
| Heavy | 13177(22.54) | 9410(22.30) | 3767(22.91) |  |

The results presented in the table are all weighted by total weights of combining sampling and matching weights (except for the frequency).

Supplementary Table 3. Comparison of baseline characteristics in participants with and without hyperlipidemia after propensity score matching.

|  | Total | Non-Hyperlipidemia | Hyperlipidemia | *P* value |
| --- | --- | --- | --- | --- |
| Age | 46.46(0.10) | 45.97(0.20) | 46.70(0.12) | 0.002 |
| Sex |  |  |  | 0.02 |
| Female | 32060(50.77) | 10242(49.67) | 21818(51.32) |  |
| Male | 31516(49.23) | 11090(50.33) | 20426(48.68) |  |
| BMI | 28.85(0.04) | 27.10(0.08) | 29.72(0.05) | < 0.0001 |
| Coffee (gram) | 306.40(2.73) | 294.43(5.56) | 312.35(3.03) | 0.005 |
| Race |  |  |  | 0.12 |
| Mexican American | 10952( 8.36) | 3448(8.44) | 7504(8.32) |  |
| Non-Hispanic Black | 14227(11.63) | 6131(12.05) | 8096(11.43) |  |
| Non-Hispanic White | 26460(66.88) | 7712(66.78) | 18748(66.93) |  |
| Others | 11937(13.12) | 4041(12.73) | 7896(13.32) |  |
| Education |  |  |  | 0.95 |
| High school or equivalent | 25597(36.75) | 8708(36.65) | 16889(36.81) |  |
| College or above | 30864(57.40) | 10776(57.48) | 20088(57.35) |  |
| Less than high school | 7115( 5.85) | 1848(5.87) | 5267(5.84) |  |
| Smoking |  |  |  | 0.01 |
| Never | 36166(55.07) | 13283(55.52) | 22883(54.84) |  |
| Former | 14640(23.63) | 3675(22.38) | 10965(24.26) |  |
| Now | 12770(21.30) | 4374(22.10) | 8396(20.90) |  |
| Alcohol consumption |  |  |  | 0.64 |
| No | 20264(25.48) | 6250(25.18) | 14014(25.63) |  |
| Mild | 30135(52.16) | 9850(52.14) | 20285(52.17) |  |
| Heavy | 13177(22.36) | 5232(22.68) | 7945(22.21) |  |

The results presented in the table are all weighted by total weights of combining sampling and matching weights (except for the frequency).

Supplementary Table 4. Comparison of baseline characteristics in participants with low and high waist circumference after propensity score matching.

|  | Total | Low WC | High WC | Pvalue |
| --- | --- | --- | --- | --- |
| Age | 46.57(0.11) | 46.89(0.16) | 46.30(0.15) | 0.01 |
| Sex |  |  |  | 0.14 |
| Female | 32060(50.84) | 10653(51.37) | 21407(50.41) |  |
| Male | 31516(49.16) | 18353(48.63) | 13163(49.59) |  |
| BMI | 28.86(0.04) | 23.68(0.03) | 33.08(0.05) | < 0.0001 |
| Coffee (gram) | 309.52(2.77) | 309.45(4.21) | 309.57(3.69) | 0.98 |
| Race |  |  |  | 0.02 |
| Mexican American | 10952( 8.13) | 4847(7.84) | 6105(8.37) |  |
| Non-Hispanic Black | 14227(11.00) | 6081(11.07) | 8146(10.95) |  |
| Non-Hispanic White | 26460(67.59) | 11413(68.23) | 15047(67.06) |  |
| Others | 11937(13.28) | 6665(12.85) | 5272(13.63) |  |
| Education |  |  |  | 0.44 |
| High school or equivalent | 25597(36.51) | 11689(36.17) | 13908(36.79) |  |
| College or above | 30864(57.87) | 14348(58.26) | 16516(57.56) |  |
| Less than high school | 7115( 5.62) | 2969(5.57) | 4146(5.65) |  |
| Smoking |  |  |  | 0.23 |
| Never | 36166(55.09) | 16730(54.75) | 19436(55.36) |  |
| Former | 14640(23.99) | 5552(24.52) | 9088(23.56) |  |
| Now | 12770(20.92) | 6724(20.74) | 6046(21.08) |  |
| Alcohol consumption |  |  |  | 0.10 |
| No | 20264(25.61) | 8260(25.51) | 12004(25.70) |  |
| Mild | 30135(52.32) | 13779(52.99) | 16356(51.77) |  |
| Heavy | 13177(22.07) | 6967(21.50) | 6210(22.53) |  |

WC: waist circumference. The results presented in the table are all weighted by total weights of combining sampling and matching weights (except for the frequency).

Supplementary Table 5. Standardized mean difference

|  | MetS | DM | Hyperlipidemia | Hypertension | WC |
| --- | --- | --- | --- | --- | --- |
| Distance | 0.047 | -0.022 | 0.088 | -0.044 | -0.045 |
| Age | 0.042 | 0.024 | 0.089 | -0.057 | 0.011 |
| Sex | 0.049 | -0.021 | -0.022 | 0.047 | 0.043 |
| Race |  |  |  |  |  |
| Mexican American | -0.002 | -0.007 | -0.001 | 0.002 | 0.004 |
| Non-Hispanic Black | -0.0001 | -0.001 | -0.0003 | 0.002 | -0.0005 |
| Non-Hispanic White | 0.004 | 0.021 | -0.008 | -0.008 | -0.016 |
| Others | -0.002 | -0.013 | 0.009 | 0.005 | 0.013 |
| Education |  |  |  |  |  |
| High school or equivalent | 0.004 | -0.009 | 0.005 | -0.016 | 0.020 |
| College or above | -0.007 | 0.011 | -0.007 | 0.014 | -0.021 |
| Less than high school | 0.003 | -0.002 | 0.002 | 0.002 | 0.001 |
| Smoking |  |  |  |  |  |
| Never | 0.011 | -0.015 | -0.009 | 0.007 | 0.007 |
| Former | 0.007 | -0.009 | 0.021 | -0.008 | -0.009 |
| Now | -0.018 | 0.024 | -0.012 | 0.001 | 0.002 |
| Alcohol consumption |  |  |  |  |  |
| No | 0.019 | -0.004 | 0.012 | -0.002 | 0.008 |
| Mild | 0.003 | -0.028 | 0.0007 | -0.021 | -0.021 |
| Heavy | -0.022 | 0.032 | -0.013 | 0.023 | 0.013 |
| Coffee (gram) | -0.073 | 0.0007 | 0.056 | -0.054 | 0.018 |

MetS: metabolic syndrome; DM: diabetes mellitus; WC: waist circumference.

Supplementary Table 6. Relationship of metabolic syndrome and its components with Parkinson's disease in stratification analysis.

| character | Metabolic syndrome | Diabetes mellitus | Hyperlipidemia | Hypertension | Waist circumference |
| --- | --- | --- | --- | --- | --- |
| Age |  |  |  |  |  |
| >50 | 0.941(0.676,1.310) | 1.238(0.845,1.815) | 0.816(0.571,1.167) | 1.281(0.910,1.803) | 0.988(0.700,1.396) |
| <=50 | 1.199(0.747,1.923) | 1.645(0.951,2.847) | 1.004(0.542,1.859) | 1.459(0.936,2.277) | **2.145(1.277,3.602)** |
| Sex |  |  |  |  |  |
| Female | 1.073(0.737,1.561) | 1.116(0.764,1.631) | 1.069(0.717,1.594) | 1.300(0.918,1.842) | 1.452(0.924,2.284) |
| Male | 0.976(0.666,1.430) | 1.684(0.993,2.855) | 0.711(0.441,1.146) | 1.377(0.884,2.144) | 0.925(0.635,1.346) |
| Race |  |  |  |  |  |
| Mexican American | 1.465(0.666,3.224) | 1.903(0.925,3.912) | 1.293(0.582,2.870) | 1.355(0.676,2.717) | 1.565(0.768,3.188) |
| Non-Hispanic Black | 0.768(0.442,1.333) | 1.596(0.799,3.187) | 0.751(0.459,1.229) | 1.414(0.825,2.424) | 1.058(0.612,1.829) |
| Non-Hispanic White | 0.986(0.710,1.371) | 1.208(0.815,1.790) | 0.903(0.618,1.320) | **1.429(1.032,1.980)** | 1.175(0.825,1.675) |
| Others | 1.484(0.679,3.246) | 2.009(0.981,4.114) | 0.902(0.410,1.982) | 0.536(0.239,1.200) | 1.994(0.982,4.050) |
| Education |  |  |  |  |  |
| High school or equivalent | 0.704(0.455,1.089) | 1.226(0.673,2.233) | 0.945(0.556,1.609) | 1.256(0.767,2.058) | 1.282(0.800,2.053) |
| College or above | 1.197(0.814,1.761) | 1.422(0.950,2.129) | 0.927(0.602,1.428) | 1.384(0.974,1.967) | 1.081(0.711,1.644) |
| Less than high school | 1.490(0.741,2.993) | 1.331(0.716,2.474) | 0.607(0.283,1.305) | 1.266(0.534,3.003) | **1.938(1.003,3.742)** |
| Smoking |  |  |  |  |  |
| never | 0.911(0.639,1.299) | 1.279(0.779,2.101) | 0.998(0.647,1.538) | 1.227(0.819,1.838) | 1.250(0.797,1.961) |
| former | 0.889(0.573,1.378) | 1.446(0.810,2.579) | 0.722(0.377,1.381) | 1.155(0.681,1.958) | 0.858(0.515,1.431) |
| now | 1.431(0.757,2.704) | 1.331(0.766,2.313) | 0.909(0.503,1.642) | 1.717(0.994,2.965) | 1.669(0.987,2.823) |
| Alcohol consumption |  |  |  |  |  |
| no | 0.965(0.647,1.439) | 1.213(0.804,1.829) | 0.738(0.449,1.213) | 1.172(0.773,1.778) | 1.175(0.788,1.752) |
| mild | 0.891(0.568,1.399) | 1.535(0.931,2.532) | 0.979(0.617,1.552) | 1.375(0.909,2.078) | 1.201(0.754,1.912) |
| heavy | 1.658(0.888,3.093) | 1.040(0.518,2.089) | 1.110(0.534,2.306) | 1.557(0.769,3.154) | 1.346(0.632,2.865) |
| Coffee |  |  |  |  |  |
| Q1 | 0.833(0.541,1.282) | 0.803(0.483,1.335) | 0.941(0.521,1.701) | 0.852(0.538,1.350) | 0.983(0.601,1.606) |
| Q2 | 0.978(0.595,1.607) | 1.380(0.829,2.298) | 0.815(0.476,1.393) | 1.168(0.724,1.883) | 1.096(0.654,1.837) |
| Q3 | 1.232(0.788,1.926) | 1.789(1.074,2.979) | 0.912(0.564,1.476) | 1.942(1.251,3.016) | 1.485(0.887,2.486) |
